# Research of Epidemic Big Data Based on Improved Deep Convolutional Neural Network

**DOI:** 10.1155/2020/3641745

**Published:** 2020-07-22

**Authors:** Wendong Wang

**Affiliations:** Yan'an University, College of Mathematics and Computer Science, Yan'an Shaanxi 716000, China

## Abstract

In recent years, with the acceleration of the aging process and the aggravation of life pressure, the proportion of chronic epidemics has gradually increased. A large amount of medical data will be generated during the hospitalization of diabetics. It will have important practical significance and social value to discover potential medical laws and valuable information among medical data. In view of this, an improved deep convolutional neural network (“CNN+” for short) algorithm was proposed to predict the changes of diabetes. Firstly, the bagging integrated classification algorithm was used instead of the output layer function of the deep CNN, which can help the improved deep CNN algorithm constructed for the data set of diabetic patients and improve the accuracy of classification. In this way, the “CNN+” algorithm can take the advantages of both the deep CNN and the bagging algorithm. On the one hand, it can extract the potential features of the data set by using the powerful feature extraction ability of deep CNN. On the other hand, the bagging integrated classification algorithm can be used for feature classification, so as to improve the classification accuracy and obtain better disease prediction effect to assist doctors in diagnosis and treatment. Experimental results show that compared with the traditional convolutional neural network and other classification algorithm, the “CNN+” model can get more reliable prediction results.

## 1. Introduction

With the arrival of the aging age and the acceleration of the pace of life, all kinds of life pressures come one by one. All these factors have caused the incidence rate of epidemic diseases, such as diabetes and cancer, which are increasing year by year. It shows that the prevention and treatment of epidemic diseases have become an urgent problem in the medical and health field. At the same time, with the continuous advancement of medical informatization, China's public health field has accumulated a wealth of data resources, which is in line with the typical characteristics of big data. The massive medical data resources usually contain a large number of valuable information, like patients' diagnosis and treatment laws. It plays an important role for us to better understand the causal relationship of epidemics and health risk factors by fully mining valuable treatment laws. As a typical chronic epidemic, the incidence rate of diabetes has remained high in recent years and shows a rising trend [1, 2]. Nowadays, the number of diabetic patients is large, and the incidence rate of this disease increases with the incubation period. Therefore, it is an urgent task to establish a reliable prediction model based on the data of diabetic patients and judge the cause of disease as early as possible.

Currently, it is common to use machine learning-related models [3–11] for disease prediction. Researchers have made many explorations in the field of diabetes diagnosis and treatment and have achieved some results [12–21]. By monitoring 16 patients, Abraham of the University of California used statistical methods to analyze the risk factors of diabetes and found that if the glycosylated hemoglobin index of diabetic patients can be obtained from 9.6% down to 7.2%, this can improve the physiological state of patients [22]. At the same time, some researchers used decision tree algorithm and multilayer perceptron to carry out comparative experiments [23]. Sneha and Gangil used machine learning methods such as random forest, SVM, *k*-means, and naive Bayes to select early attributes that can be used to predict diabetes. The results show that the decision tree algorithm and random forest can achieve the best prediction effect for diabetes data [24]. Meanwhile, deep convolutional neural network is a hot research field in recent years. Because of its powerful feature extraction ability, it can mine deeper features from a large number of training data with the hierarchical network structure, so as to extract the feature information that cannot be obtained by traditional classifiers. Therefore, it has been widely used in speech recognition, image recognition, text detection, and so on [18, 25–33]. As we know, the medical data set has the characteristics of large amount of data and rich features, so it is helpful to discover potential medical laws and valuable information among medical data by applying deep convolutional neural network to medical data. In a word, it will have important practical significance and social value [12, 34–39]. For example, Swapna et al. applied CNN to realize automatic detection of diabetes mellitus [14]. They used heart rate variability data to obtain heart rate signals and used CNN-LSTM combined network to carry out automatic anomaly detection and fully connected structure, which can realize automatic detection and accurate diagnosis of diabetes [13].

In summary, although researchers have done a lot of research on diabetes, most of them pay attention to the diagnosis method, blood glucose detection method, and complications of diabetes. Few researches are aimed at the inpatients of diabetes. In view of this, a typical epidemic disease, like diabetes mellitus, is taken as an example. And an improved algorithm based on the deep convolutional neural network is proposed to predict the change of diabetes based on the data of inpatient medical records of diabetes patients. The improved algorithm takes advantages of both the bagging integrated classification algorithm and the deep convolutional neural network. It not only has the good data classification ability but also has the strong feature extraction ability, which can effectively improve the classification accuracy and obtain better disease prediction effect to assist doctors in diagnosis and treatment. The innovation of this paper is that the bagging integrated classification algorithm is applied instead of the output layer function of the deep convolutional neural network to build an improved deep CNN algorithm (“CNN+” for short) for the data set of diabetic patients. On the other hand, in order to ensure that the experimental results and analysis are based on good structured data, data preprocessing is also carried out in the process of the experiment, including data cleaning, data balancing, data feature processing, and abnormal data processing. Finally, the structure of the paper is as follows.

Part 1 is the introduction to the research background, status, and significance of the thesis.

Part 2 explains the relevant work of the thesis, so as to provide a theoretical basis for the follow-up research.

Part 3 introduces the principle of the improved algorithm proposed in this paper in detail.

Part 4 verifies the effectiveness of the method proposed in this paper in the detection of diabetes data based on a series of experiments.

## 2. Related Work

Deep convolutional neural network is a special type of neural network. Its super learning ability is mainly achieved by using multiple nonlinear feature extraction, which can automatically learn hierarchical representation from data. CNN is a deep neural network including input layer, convolution layer, pooling layer, full connection layer, and output layer. Firstly, CNN extracts the features of input data by convolution. Then, by using weight sharing and pooling, the difficulty of training network and the redundant data are greatly reduced, and the features are retained to the maximum extent. Finally, features are transferred from the full connection layer to the output layer for classification [29]. Because of convolution and pooling operation, the deep CNN has the characteristics of sparse connection, parameter sharing, translation, and local translation invariance, which can effectively extract image features while reducing learning parameters and training difficulty, making CNN widely used.

### 2.1. The Main Components of Deep CNN

**Figure 1:**
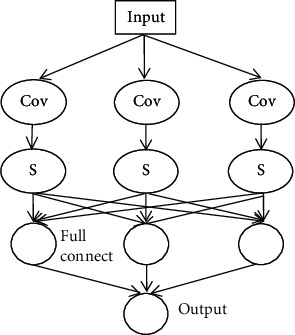
The structure of the convolutional neural network.

A typical deep convolutional neural network is generally composed of input layer, convolution layer, pooling layer, full connection layer, and output layer. Its structure is shown in Figure 1. The difference between convolutional neural network and traditional neural network is that the convolutional neural network contains a feature extractor composed of convolution layer and pooling layer. Then, we briefly introduced the function of these components in CNN.

**Figure 2:**
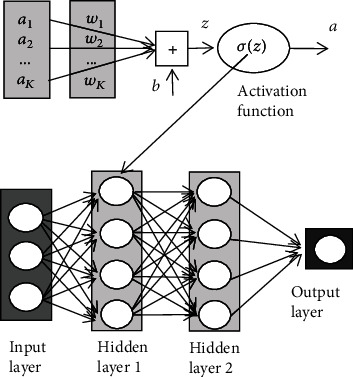
The operation diagram of the convolution layer.

**Figure 3:**
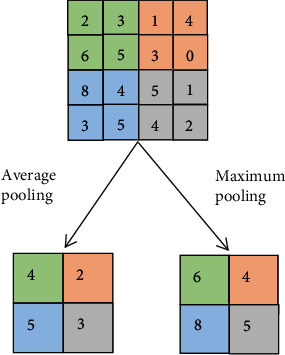
The pooling methods.

1. Deep CNN can directly take many kinds of data as the input data, such as image and audio. But in order to get better results, it is usually necessary to preprocess these data
2. The convolution layer is the key section of the convolutional neural network. It can convolute the input data to extract the features and transmit the convoluted results to the lower layer. The essence of convolution is to represent the input in another way. If the convolution layer is regarded as a black box, then we can regard the output as another representation of the input, while the training of the whole network is to train the parameters needed for this representation.  Figure 2 is a diagram of the convolution. In Figure 2, *w* and *b* are the parameters needed for network training. In the convolution layer, we need to apply activation function to nonlinear operation. The deep convolution network connects series of the small neural networks so as to form the deep neural network, which is mainly realized by local receptive field and weight sharing. The former refers that the neuron is only connected with its adjacent upper layer neuron, and the final global feature is formed by combining the learned local features. Weight sharing means that the same convolution kernel uses the same weight parameter when it operates on different local receptive fields. In this way, the connection between network layers can be reduced, so as to reduce the amount of parameter calculation in the process of network training
3. The pooling layer is also known as subsampling, which is a special data processing operation in the convolutional neural network. After the convolution operation, the data needs to be further processed by pooling to reduce the dimension of the extracted features. In this way, it can effectively remove the problem of large amount of calculation.  Figure 3 shows the commonly used pooling method. Because the maximum pooling is achieved by extracting the point with the maximum value of local area, which has the advantage of retaining the factor with the largest influence in the feature area and effectively avoiding the information loss, we use the maximum pooling in the pooling layer
4. The full connection layer is usually used for classification at the end of the network. Unlike pooling and convolution, it is a global operation. Each node of the full connection layer is connected with all nodes of the previous layer, which is used to integrate the extracted features and transmit the signals to other full connection layers. Because of its all connected characteristics, the parameters of the general all connected layer are more, which is the layer consuming the most parameters in the network. Therefore, there are many parameters in the full connection layer, which consumes the most energy in the network
5. The output layer of the deep convolutional neural network completes different work according to the purpose of research. It usually uses the softmax function to calculate the classification results

### 2.2. The Derivation of Deep CCN

The essence of deep CNN is a mapping between input and output. It can learn a lot of mapping relations between input and output without any precise mathematical expression. As long as the convolution network is trained with known patterns, the network has the mapping ability between input and output pairs. The parameters of the deep convolutional neural network are defined as follows:

Let *L* be the number of network layers. In the convolution layer, the size of the convolution kernel is *K*. The dimension of the convolution kernel matrix is defined as *F*. *p* represents the filling size, and the steps of convolution kernel moving are *S* [28]. Before the data is transferred to the convolution layer, it is necessary to fill in missing data. Suppose the input data after filling is *a*^1^. Then, the solution of deep CNN is as follows:

(1) Initialize parameters
Initialize the weight parameters *W*, the bias *b* of the network, the maximum number of iterations *T*, and the iteration threshold *ε*.
(2) Training phase
*Step 1* (forward propagation). Select the training set and input them into the network. Calculate the corresponding output.
For *l* = 2 to *L*-1:
If *l* is a convolution layer, then
  (1) $$\begin{array}{c} a^{l}=\text{RELU}\left(z^{l}\right)=\text{RELU}\left(a^{l}\times W^{l}+b^{l}\right). \end{array}$$
If *l* is a pool layer, then
  (2) $$\begin{array}{c} a^{l}=\text{pool}\left(a^{l-1}\right). \end{array}$$
If *l* is a full connection layer, then
  (3) $$\begin{array}{c} a^{l}=\sigma \left(z^{l}\right)=\sigma \left(W^{l}a^{l-1}+b^{l}\right). \end{array}$$
End for.
Finally, the output layer *L*
  (4) $$\begin{array}{c} a^{L}=\text{softmax}\left(z^{L}\right)=\text{softmax}\left(W^{L}a^{L-1}+b^{L}\right). \end{array}$$
*Step 2* (backward propagation). Calculate the error between the actual output and the corresponding ideal output.
For *l* = *L* − 1 to 2:
If *l* is a full connection layer, then
  (5) $$\begin{array}{c} \delta^{i,l}={\left(W^{l+1}\right)}^{T}\delta^{i,l+1}\Theta \sigma \left(z^{i,l}\right). \end{array}$$
If *l* is a convolution layer, then
  (6) $$\begin{array}{c} \delta^{i,l}=\delta^{i,l+1}*\text{rot}180\left(W^{l+1}\right)\Theta \sigma \left(z^{i,l}\right). \end{array}$$
If *l* is a pool layer, then
  (7) $$\begin{array}{c} \delta^{i,l}=\text{upsample}\left(\delta^{i,l+1}\right)\Theta \sigma \left(z^{i,l}\right). \end{array}$$
End for.
*Step 3* (update weights and bias). Adjust the weight matrix and bias according to the method of minimizing error.
For *l* = 2 to *L*:
If *l* is a full connection layer, then
  (8) $$\begin{array}{c} W^{l}=W^{l}-\alpha \overset{m}{\underset{i=1}{\sum }}\delta^{i,l}{\left(a^{i,l-1}\right)}^{\text{T}},\\ b^{l}=b^{l}-\alpha \overset{m}{\underset{i-1}{\sum }}\delta^{i,l}. \end{array}$$
If *l* is a convolution layer, then
  (9) $$\begin{array}{c} W^{l}=W^{l}-\alpha \overset{m}{\underset{i=1}{\sum }}\delta^{i,l}*a^{i,l-1},\\ b^{l}=b^{l}-\alpha \overset{m}{\underset{i-1}{\sum }}\underset{\mu ,\nu }{\sum }{\left(\delta^{i,l}\right)}_{\mu ,v}. \end{array}$$
End for.
*Step 4*. If ‖*a*^(*t* + 1)^ − *a*^(*t*)^‖ < *ε* or *t* > *T*, the loop ends. Otherwise, return to Step 1.
(3) Output
Output the relation coefficient matrix *W* and bias *b*.

## 3. The Improved Deep Convolutional Neural Network

The deep convolutional neural network mainly relies on the convolution layer and the pooling layer for feature extraction and feature selection and uses the full connection layer for feature integration and the output layer for feature classification. In order to further improve the classification performance of the deep CNN model, this paper proposes to use the bagging integrated classification algorithm with better classification performance to replace the output layer function of the traditional convolutional neural network, so as to further optimize the classification ability of the convolutional neural network. The principle of the improved algorithm is to use the deep convolutional neural network for training and then extract the features integrated in the full connection layer as a new data set. Then, it will be trained as the input data of the bagging ensemble learning classifier. Finally, output the classification result based on the voting method. In other words, in the improved deep convolutional neural network, the softmax function of the output layer will be replaced by the bagging algorithm. In this way, we can not only use the convolutional neural network to extract the potential features of the data set but also use integrated learning to classify the features, so as to achieve a good effect of disease prediction.

### 3.1. The Bagging Integration Classification

As we know, the bagging method is a kind of parallel integrated classification algorithm which randomly selects training sets. The advantage of the bagging method is that it introduces bootstrap sampling in the training process. In this way, it can ensure that the training subsets are as independent as possible, so as to enhance the differences between the classifiers. Because these basic classifiers can analyze the features of the data set from different angles, they can reduce the generalization error of the algorithm and improve the classification accuracy. Moreover, compared with the ordinary single classifier, they can achieve better classification effect. At the same time, every training in the bagging can run in parallel, which greatly shortens the running time and improves the classification efficiency. What is more, the proportion of noise data is usually relatively low in the practical application, so the interference of the noise data on the final classification results can be effectively alleviated by means of multiple random sampling of the bagging method [40].

**Figure 4:**
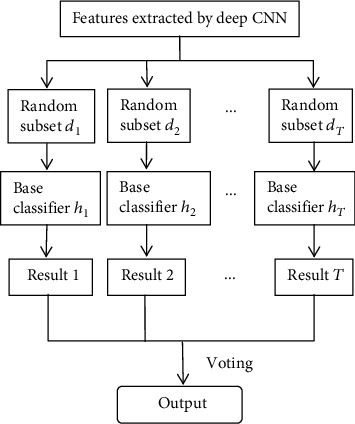
The structure of the integrated learning layer.

In summary, the structure of the integrated learning layer is shown in Figure 4.

Meanwhile, define *Y* = {−1, +1} as a classification label set. *T* represents the number of the base classifiers. Then, the assumed function of the bagging method is

(10) $$\begin{array}{c} H\left(x\right)=\mathrm{sign}\left(\overset{T}{\underset{i=1}{\sum }}h_{i}\left(x\right)\right). \end{array}$$

The specific steps of the bagging algorithm are described in Algorithm 1.

### 3.2. The Deep CNN Based on the Bagging Algorithm

**Figure 5:**
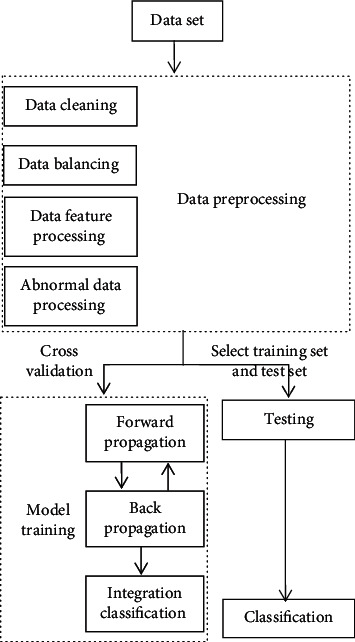
The flow of the improved deep CNN algorithm.

In the training stage of the deep CNN, the amount of data used is relatively large compared with the number of features, so there is no need to worry about the fitting problem of the network and no need to drop out the network. Firstly, input the data into the deep CNN model for training, and save the trained model which can achieve the best classification effect. The features integrated by the full connection layer of the saved model are the best features. Then, the best features saved are used as the input data of the integrated learning layer for classification.  Figure 5 shows the flow of the improved deep CNN algorithm.

As shown in Figure 5, data cleaning, data balancing, data feature processing, and exception data processing can be collectively referred to as data preprocessing, so that the whole algorithm process can be divided into data preprocessing and training model. Because the data set used in this experiment is the original data recorded by the hospital, the data resources cannot be trained directly due to some irresistible factors. Firstly, we need to analyze the data and then clean the data set properly. Then, according to the fact that the distribution of medical data is unbalanced in real life, we need to balance the data after cleaning. After the data is balanced, the next step is to analyze the data features in the data set, obtain high-quality feature attributes, and process the abnormal data. Finally, put the preprocessed data input into the deep DNN for training, and finally, get the classification result of the trained data based on the integrated classification layer. The steps of the improved deep CNN are described in detail as follows.

1. *Data Cleaning*. This deals with the missing values in the original data set. It mainly solves the problems of data format inconsistency, data incompleteness, data record error, and so on.
2. *Data Balancing*. The data set chooses from the medical data in real life. Due to abnormal data, data value missing, and other reasons, the data after cleaning still has the problem of unbalanced distribution. In view of this, we use a few kinds of oversampling technology to process the data set, so that the sample data can reach equilibrium and ensure the accuracy of the experiment and the value of the experimental results.
3. *Data Feature Processing*. In this study, the embedding method is used to select features. We mainly use the xgboost algorithm to sort each feature according to its importance. At the same time, considering the opinions of diabetes experts, we screened out the important characteristics finally.
4. *Abnormal Data Processing*. Because the data set is manually recorded by the medical staff in the hospital, there are some noise data. In order to avoid the impact of these noise data on the experimental results, it is necessary to detect the outliers of these data and screen out the abnormal data. In the paper, the isolated forest algorithm is used to detect outliers, so as to obtain a smoother data set.
5. *Model Training*. Through the above processing, we can get the data set with balanced, smooth, and high feature contribution and use it as the input data of DNN for model training.
6. *Classification Training*. The trained data feature is used as the new input data of the bagging algorithm in the integrated classification layer to train the base classifiers.
7. *Output Results*. Finally, we will have tests on the improved deep DNN and output the classification results.

Algorithm 2 describes the detailed description of the deep CNN based on the bagging algorithm.

## 4. Experimental Studies

### 4.1. The Experimental Design

The experimental environment adopts Intel (R) core (TM) i7-9700u processor, 3.0 GHz main frequency, 8 G memory, and Windows 10 operating system. We use PyCharm 2018.1.4 (Professional Edition) as the experimental platform and python 3.6 as the coding tool. The algorithm is realized by building the tensor flow machine learning library for programming.

### 4.2. The Experimental Data and Parameter Setting

In this study, we adopt the data of diabetes patients admitted to 130 hospitals in the United States for 10 years as the experimental data [41, 42]. There are 10766 samples in the data set. And each sample contains 49 attribute columns and 1 label variable. The label variable 1 indicates that the patient is readmitted within 30 days, and 0 indicates that the patient is not readmitted within 30 days after discharge. The 49 attributes include personal information (such as patient number, race, gender, and age), diagnosis details (such as admission type, outpatient diagnosis record), medication records (such as medication quantity and diabetes drugs), and examination records. Therefore, the data set has rich samples and complete characteristics on the diagnosis and treatment of diabetes. As the data of diabetes used in this study have only 36 features after data preprocessing, we limit the depth of convolutional neural network. This is different from the traditional convolutional neural network with deep network structure for image processing. In addition, we have also made customization in the design of convolution core and pool filter, and its structural parameters are shown in Table 1.

### 4.3. Experimental Results and Analysis

In the process of experimental analysis, we usually judge the performance of classifier based on the accuracy of classification results. However, for some application scenarios, accuracy as an evaluation index has certain limitations, and it cannot always effectively evaluate the work of a classifier. In view of this, we need to introduce other indicators to evaluate the experimental results. Therefore, this paper uses accuracy, recall, precision, and F-measure to evaluate the performance of the improved deep CNN. Then, each indicator is defined as follows:

(11) $$\begin{array}{c} \text{accuracy}=\frac{\left(\text{TP}+\text{TN}\right)}{\left(P+N\right)},\\ \text{recall}=\frac{\text{TP}}{P},\\ \text{precision}=\frac{\text{TP}}{\left(\text{TP}+\text{FP}\right)},\\ \text{F}‐\text{measure}=\frac{2\text{TP}}{\left(2\text{TP}+\text{FP}+\text{FN}\right)}, \end{array}$$

where *P* is the number of samples identified as positive in the test set, TP is the number of samples correctly predicted as positive by the classifier, and FP is the number of samples incorrectly predicted as negative by the classifier. F-measure is the harmonic average of recall rate and precision, and it is a comprehensive index that can better reflect the classification performance. In the experiments based on the medical data, researchers pay more attention to the recall rate than the accuracy. In this experiment, if the recall rate is higher, it can show that the possibility of judging patients' bad recovery as good recovery is lower. And if the prediction is more accurate, it is more helpful to achieve the experimental goal.

**Figure 6:**
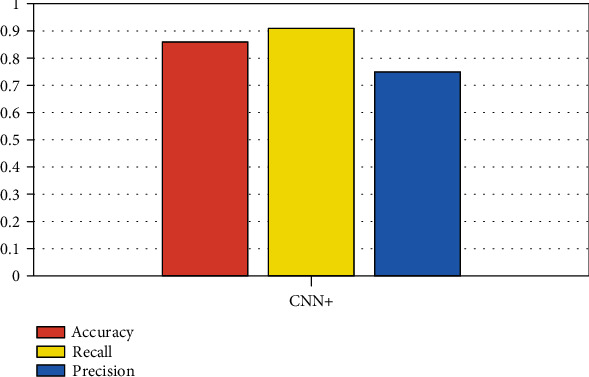
The application of CNN+ based on the data of diabetic medical records.

After completing the above experimental parameter settings and experimental index settings, firstly, we trained the data set with the deep CNN. And in the training, we set the number of iterations as 1000 and then save the parameters of the model. Then, we extract the features of CNN in the full connection layer and segmented the data. Then, we use the bagging integrated classification algorithm to train the feature data extracted from the full connection layer and use the voting method to vote the experimental results, so as to obtain the classification results. As in the paper, we use recall, precision, and accuracy to measure the effect of classification; Figure 6 shows the variance and average of the above three indicators.

**Figure 7:**
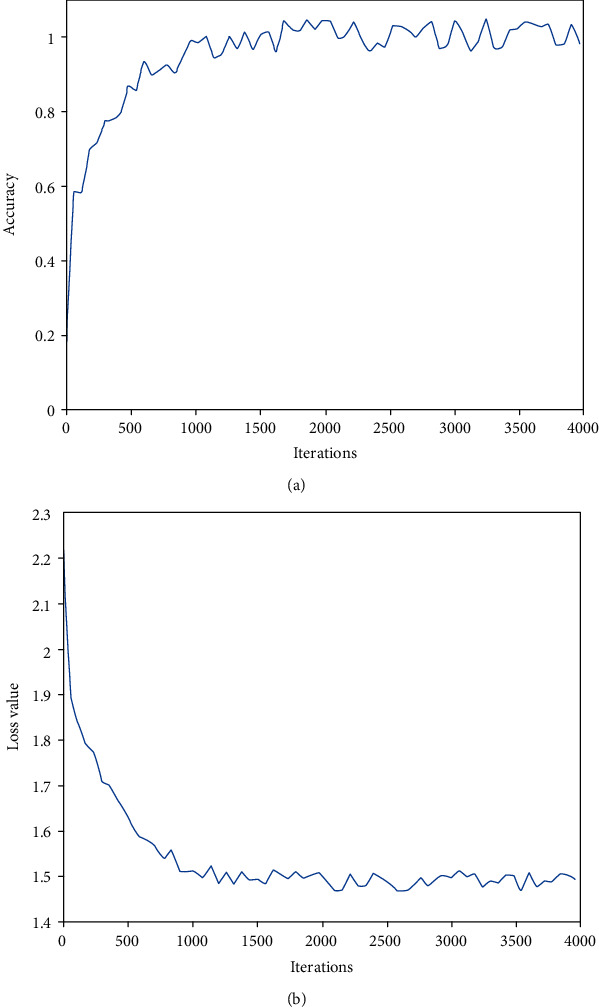
The curve of accuracy and loss value with iteration times.

In order to verify the effectiveness of the algorithm in this paper, we have carried out 4000 iterations of CNN+, and Figure 7 shows the curve of the accuracy and the loss of network training with the number of iterations.

As seen in Figure 7(a), the training accuracy gradually increases with the number of iterations and then tends to be stable. It can be seen from Figure 7(b) that the loss value gradually decreases with the increase of the number of iterations and then starts to stabilize near the smaller value, indicating that the trained network model has better stability.

Finally, in order to verify the necessity of data balance processing for the original data in the process of the experiment, we carry out the experiment based on the unbalanced data and balanced data. Meanwhile, the compared experiments were conducted among the traditional convolutional neural network and decision tree, random forest, and naive Bayes. The experimental results are illustrated in Tables 2 and 3, respectively.

As seen from Tables 2 and 3, we can find the following information:

1. Whether in the balanced data set or in the nonbalanced data set, CNN+ can achieve better experimental results in general. Compared with the experimental results of CNN, decision tree, random forest, and naive Bayes classifier, the CNN+ accuracy, recall rate, and precision are significantly improved. It shows that the CNN+ algorithm has higher classification accuracy and better stability
2. In addition, comparing the experimental results of Tables 2 and 3, it can be found that using the data set after data equalization processing is indeed helpful to improve the classification accuracy of the classifier. Comparing the experimental results of the above algorithms, we can see that the experimental results on the balanced data set are obviously better than those on the unbalanced data set
3. In summary, the prediction stability of the algorithm is as important as the prediction accuracy in the field of medical research, which is related to the patient's condition recovery. The improved deep convolutional neural network can achieve good accuracy and precision in the detection of diabetes data. And it is easy to learn and train, has high stability, and shows certain advantages compared with other classifiers. Therefore, it provides the possibility for its application in practice

At the same time, we also compared the research results of other researchers based on this data set. For example, Strack et al. also conducted experiments on the same data set [42]. They used HbA1c, or glycosylated hemoglobin, as an indicator to predict the probability of readmission of patients with diabetes after treatment. In the study, the researchers also noticed that few researchers studied diabetes care during hospitalization, so they proposed to use the data set of inpatient care records to predict the relationship between the patients' readmission in the short term. The key of their study was to use multivariate logistic regression to fit the relationship between HbA1c value and the short-term readmission of patients. However, due to the small sample size of HbA1c value in this data set, the results of this study only prove that there is a relationship between HbA1c value and the probability of readmission. Compared with the study of Strack et al., we considered a number of admission records. Firstly, we use the deep convolutional neural network to extract features and make full use of the useful features in the data set. At the same time, we combine with the bagging integrated method for classification, and the reliable prediction effect is achieved.

## 5. Conclusion

With the increasing rate of epidemic disease (like cancer and diabetes), the human health has been seriously threatened, so it is necessary to study the prevention and treatment of epidemic diseases. In view of this, the deep learning technology was used to analyze the data of diabetes inpatient cases, which can help mine valuable treatment rules from them and assist doctors in diagnosis and treatment and improve treatment efficiency. Due to the large number of features and large amount of data in this data set, an improved deep convolutional neural network algorithm is proposed to predict the condition of diabetes, so as to improve the classification accuracy of deep CNN. The improved deep CNN can take the advantages of the bagging integrated classification algorithm based on the deep CNN. In order to demonstrate the effectiveness of the improved algorithm, the comparative experiments were conducted among the traditional neural network and classifiers. The experimental results prove that the improved algorithm does improve the classification accuracy and stability. Because of the large sample size of the data set, good experimental results can be achieved in the convolutional neural network. However, the convolutional neural network requires a large amount of data and data characteristics; the experimental results for the small sample data set are not ideal. The classification of small samples is worth further study.

## Figures and Tables

**Algorithm 1:**
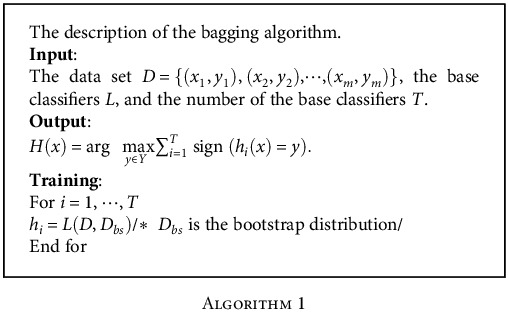


**Algorithm 2:**
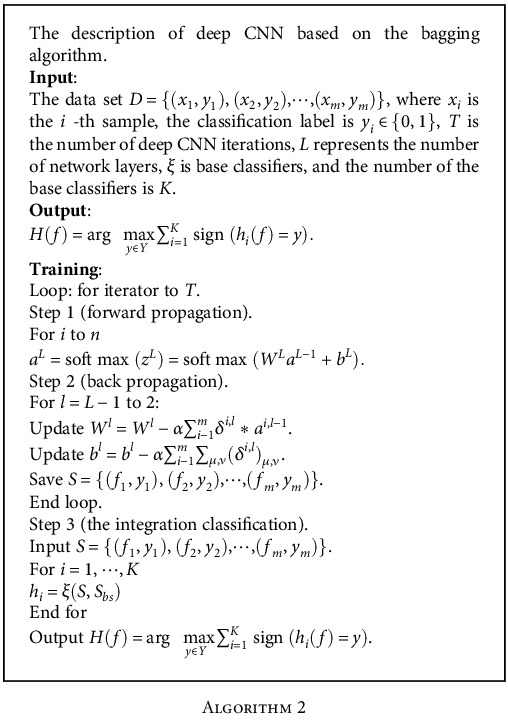


**Table 1:** The parameter settings of different network layers.

Network layer	Input	Filter	Step	Padding
Input layer	6∗6∗1	/	/	/
Convolution layer 1	6∗6∗1	3∗3∗32	1	Same
Pool layer 1	6∗6∗32	2∗2	1	Valid
Convolution layer 2	5∗5∗32	3∗3∗64	1	Same
Pool layer 2	5∗5∗64	2∗2	1	Valid
Full connection layer 1	128	/	/	/
Full connection layer 2	128	/	/	/

**Table 2:** The segmentation accuracy of brain tissue based on various algorithms.

Models	Accuracy	Recall	Precision	F-measure
Decision tree	0.81	0.85	0.70	0.77
Random tree	0.80	0.83	0.71	0.77
Naive Bayes	0.78	0.81	0.70	0.75
CNN	0.81	0.84	0.73	0.78
CNN+	0.83	0.88	0.73	0.79

**Table 3:** The segmentation accuracy of brain tissue based on various algorithms.

Models	Accuracy	Recall	Precision	F-measure
Decision tree	0.82	0.86	0.72	0.78
Random tree	0.82	0.81	0.72	0.77
Naive Bayes	0.80	0.82	0.73	0.78
CNN	0.83	0.90	0.72	0.80
CNN+	0.84	0.91	0.74	0.81

## Data Availability

All relevant data are within the paper.
